# Pregnancies in patients with systemic lupus erythematosus during 2000–2018 in Finland: a case–control study

**DOI:** 10.1007/s00296-024-05564-x

**Published:** 2024-04-02

**Authors:** Pia Elfving, Simo Kariniemi, Hannu Kautiainen, Vappu Rantalaiho, Lauri J. Virta, Kari Puolakka, Merja K. Laine

**Affiliations:** 1 Department of Medicine, Kuopio University Hospital, P.O. Box 100, 70029 KYS Kuopio, Finland; 2 Institute of Clinical Medicine, University of Eastern Finland, Kuopio, Finland; 3 School of Medicine, University of Eastern Finland, Kuopio, Finland; 4 Jyväskylä Central Hospital, Jyväskylä, Finland; 5 Primary Health Care Unit, Kuopio University Hospital, Kuopio, Finland; 6 Folkhälsan Research Centre, Helsinki, Finland; 7 Faculty of Medicine and Health Technology, Tampere University, Tampere, Finland; 8 Centre for Rheumatic Diseases, Tampere University Hospital, Tampere, Finland; 9 Kanta-Häme Central Hospital, Hämeenlinna, Finland; 10 Research Department, Social Insurance Institution, Helsinki, Finland; 11 Terveystalo Healthcare, Lappeenranta, Finland; 12 Department of General Practice and Primary Health Care, University of Helsinki and Helsinki University Hospital, Helsinki, Finland

**Keywords:** Pregnancy, Systemic lupus erythematosus, Epidemiology, Congenital abnormalities

## Abstract

**Objectives:**

The aim was to investigate, how pregnancies proceed in patients with systemic lupus erythematosus (SLE) compared to their individually matched population controls.

**Material and methods:**

Adult incident SLE patients were identified from the register of new special reimbursement decisions for SLE drugs in 2000–2014. For each patient, 1–3 randomly selected controls from the Population Register Centre were matched. Data regarding pregnancies were obtained from the Finnish Medical Birth Register, Care Register and Register of Congenital Malformations until 2018. The study utilized data from the Drug Purchase Register and educational information from Statistic Finland.

**Results:**

A total of 163 deliveries for 103 mothers with SLE and 580 deliveries for 371 population controls were identified. The duration of pregnancies in SLE women was significantly shorter compared to controls (38.9 versus 39.6 weeks). There were more urgent Caesarean Sections. (15% versus 9%) and need for care at neonatal intensive care unit (NICU) (21% versus 11%) among deliveries in SLE mothers. No statistical difference was observed between SLE and control groups in the occurrence of preeclampsia or major congenital malformations. Gestational age was 2.5 weeks shorter when the mother experienced pre-eclampsia. Hydroxychloroquine was purchased by 30% of SLE mothers during pregnancy.

**Conclusion:**

The course of pregnancies in Finnish SLE patients seems to be quite moderate compared to controls, and no new safety issues were detected. The low utilization of hydroxychloroquine indicates that the benefits of the drug to pregnancy and disease course are not optimally recognized by specialists treating SLE mothers.

## Introduction

The onset of systemic lupus erythematosus (SLE) is often during patients’ reproductive years, and pregnancies in female SLE patients are common. Therefore, many potential threats exist regarding family planning. Of particular concern is active disease, especially renal manifestations, during pregnancy [1, 2]. Results from earlier studies demonstrate a strong association between antiphospholipids and incidence of thrombotic events and an adverse course of pregnancy [2–4], while SSA and SSB antibodies seem to predispose foetus to congenital cardiac conduction defects quite rarely, in 1–2% of the cases [5–7]. As a general practice, it is recommended that in every SLE patient risk factors affecting pregnancy should be individually evaluated and the disease itself should be at least 6 months in remission before conception [8].

Another major issue in SLE pregnancies is medication. Pregnant women tend to have concerns about the possible harmful effects of medication on the foetus. This may result in a lack of adherence to medication [9]. Hydroxychloroquine (HCQ), azathioprine, and if needed cyclosporine and tacrolimus are commonly allowed anti-rheumatic medications during pregnancy. Instead, contraindicated drugs during pregnancy are mycophenolate mofetil, methotrexate, leflunomide, cyclophosphamide, belimumab, rituximab and anifrolumab. However, in a critical situation, cyclophosphamide can be used as a life-saving treatment regardless of the consequences for the foetus [8, 10]. There is an urgent need to address the gestational safety concerns caused by SLE and drug therapy with careful family planning [10]. According to international guidelines, a shared decision-making process is recommended [11–13]. Pregnancy planning should be started well in advance to achieve and maintain remission by potentially changing the drug therapy [11].

Despite the right timing and deliberate planning, problems can still occur. Evidence from previous studies has shown that pre-eclampsia, small for gestational age and intrauterine growth retardation are more common in SLE pregnancies than in the control population [2, 14–16]. Additionally, stillbirths occur more often in SLE pregnancies [15, 16], and at least one recent study reported increased maternal mortality among SLE mothers [17]. A theoretical framework behind these difficulties is not yet fully understood, but it is complex and individual in nature, just like the disease itself [18].

The aim of this study was to estimate whether there is a difference in the duration of pregnancy, amount of pre-eclampsia, urgent Caesarean sections and need for care at neonatal intensive care units (NICU) in SLE pregnancies compared to their population controls.

Secondary objectives were to analyse the amount of congenital malformations in infants born to SLE mothers compared to those born to control mothers and in addition clarify the use of hydroxychloroquine during SLE pregnancies in Finland.

## Patients and methods

The study population consists of all 845 adult female patients (age > 17 years), for whom a special reimbursement for SLE medication had been applied at the time of diagnosis by a specialist treating the disease between 1 January 2000 and 31 December 2014. The patients were identified from the national special reimbursement decision register by the International Classification of Disease 10th revision (ICD–10) code for SLE (M32). The register is administrated by the Social Insurance Institution. The date of awarding of compensation was defined as an index date (ID) for the patient and her controls. For each patient, one to three population controls matched according to age, sex, and place of residence at the ID were randomly selected from the Population Register Centre. The detailed identification method is explained in more detail in a previous study [19]. Any clinical data, or data regarding whether the patients fulfilled the classification criteria for SLE were not available. Data on educational attainment were obtained from Statistics Finland, and education level was categorized as basic, secondary and high level according to the number of years of schooling. The incident SLE patients and their controls were linked to the Finnish Medical Birth Register, which is maintained by the National Institute for Health and Welfare (THL). Shortly, the study was nationwide and register based, and it included all adult incident SLE females, who were granted a special reimbursement for SLE medication in 2000–2014 and gave birth during the follow-up period by the end of 2018.

The birth register receives data on maternal and infant’s health records from all deliveries in Finland hospitals, and it contains home births as well. The study included data on those mothers who had a SLE diagnosis at the time of pregnancy (years 2000–2018). The maternal data on deliveries were retrieved as follows: mother’s age, height and pre-gestational weight, time since ID, cohabiting, order of the pregnancy, body mass index (BMI = body weight divided by height^2^, kg/m^2^), smoking and the best estimate of gestational age according to the five class scale (pregnancy weeks < 28, 28–31.9, 32.0–33.9, 34.0–36.9 and ≥ 37).

The following information concerning pregnancies was also gathered from the register: mode of delivery, infant’s sex, infant’s birthweight and body length, ponderal index (infant’s body weight divided by body length^3^, kg/m^3^), one minute Apgar scores, number of the infants, need of neonatal intensive care unit (NICU) treatment before age of seven days, and need of antibiotic treatments before age of seven days. By Finnish law, all service providers are obligated to produce data on all outpatient visits in specialized care and hospitalizations. These data are gathered in the Care Register of the THL. The following information with the ICD-10 codes was gathered from both the Finnish Medical Birth Register and the Care Register, covering the same observation time: gestational pregnancy-induced hypertension without significant proteinuria (O13), pre-eclampsia (O14) and eclampsia (O15).

In addition, the reimbursed and purchased medications across the pregnancy were clarified through their Anatomical Therapeutic Chemical (ATC) codes from the Drug Purchase Register maintained by the Social Insurance Institution. The retrieved medical information included anti-rheumatic medication (ATC codes hydroxychloroquine P01BA02, glucocorticoids H02, azathioprine L04AX01, cyclosporine L04AD, and sulfasalazine A07EC01) and medication for blood pressure (ATC codes other hypertensives C02, diuretics C03, peripheral vasodilators C04, beta-blockers C07, calcium-channel blockers C08 and agents acting on the renin-angiotensin system C09). A drug was interpreted to be used if an individual had purchased it at least once during the pregnancy.

Lastly, the pregnant SLE patients and their controls were linked to the Register of Congenital Malformations, which is also maintained by the THL. It covers all the data on major congenital malformations with their ICD-10-codes after 22 weeks of gestation up to one year after the delivery.

Permissions to use databases were obtained from each registered holder. Since the study was register-based and data were analysed pseudo anonymously without contacting study participants, neither approval of the ethics committee nor the patients’ informed consent was required by Finnish law.

### Statistical methods

The results were presented as means with standard deviations (SD) and as counts with percentages. Statistical comparisons between SLE and control groups were done using the chi-square test and t-test. Generalized estimating equations (GEE) model with the exchangeable correlation (appropriate distribution and link function) analyses were used to account for correlations between repeated pregnancies in the same woman. Normal distributions were evaluated graphically using the Shapiro–Wilk W test. Stata 17.0 (StataCorp LP, College Station, TX, USA) was used for the analysis.

## Results

A total of 163 deliveries for 103 SLE mothers were identified during the follow-up time. At the same time, 371 population controls had 580 deliveries. The basic characteristics of the pregnancies are summarized in Table 1. The age distribution was similar for both groups (*p* = 0.87). A higher percentage of SLE women (45%) were primiparous compared to the controls (36%) (*p* = 0.036).

**Table 1:** Characteristics of the pregnancies in 2000–2014 of diagnosed SLE patients and their population controls in Finland

Characteristics of pregnancies	SLE *n* = 163	Controls *n* = 580	*p* value
Mothers, *n*	103	371	
Cohabiting, *n* (%)	152 (93)	528 (91)	0.37
Primipara, *n* (%)	74 (45)	211 (36)	0.036
Mother’s age at delivery, mean (SD)	31.2 (4.5)	31.3 (4.7)	0.85
Time since index date, months, mean (SD)	6.2 (3.4)	5.7 (3.6)	0.091
Pre-pregnancy weight, kg, mean (SD)	65.6 (10.6)	67.2 (14.1)	0.21
Height, cm, mean (SD)	166 (6)	166 (6)	0.71
Pre-pregnancy body mass index, kg/m^2^, mean (SD)	23.8 (3.8)	24.4 (4.8)	0.18
< 18.5, *n* (%)	2 (1)	11 (2)	
18.5–24.9, *n* (%)	101 (69)	329 (64)	
25.0–29.9, *n* (%)	31 (21)	116 (22)	
≥ 30, *n* (%)	13 (9)	62 (12)	
Smokers, *n* (%)			0.28
No	140 (89)	490 (87)	
Quit during the first trimester	10 (6)	25 (4)	
Continued after the first trimester	8 (5)	46 (8)	
Education level, *n* (%)			0.13
Basic	67 (41)	272 (47)	
Secondary	53 (33)	183 (32)	
High	43 (26)	125 (22)	

*SLE* systemic lupus erythematosus, *n *number, *% *percentage, *SD *standard deviation, *index date *the date of special reimbursement decision for SLE medication in patients (equal with the inclusion of controls), *kg* kilograms, *cm* centimetre, *m*^*2*^ square metre

The outcomes of the deliveries are shown in Table 2. The duration of pregnancy in SLE women was significantly shorter than in the controls. In both groups, there were a few sporadic births at the early stages of pregnancy. In both SLE and control groups, the pregnancies were a mean 2.5 weeks shorter, when pre-eclampsia was present. There was no statistical difference in the occurrence of pre-eclampsia between groups. There were more urgent Caesarean sections in SLE pregnancies than in the controls (15% versus 9%). Additionally, there was a higher need for NICU treatment among deliveries in SLE mothers (21% versus 11%). Female infants born to SLE mothers had a smaller birth weight and body length than those of the control mothers, but the difference was not significant after considering the gestational age.

**Table 2:** Outcomes of the deliveries in 2000–2014 of diagnosed SLE patients and their population controls in Finland

Outcomes of the deliveries	SLE *n* = 163	Controls *n* = 580	*p* value
Number of infants, *n* (%)			0.73
Singleton	160 (98)	571 (98)	
Twins	3 (2)	9 (2)	
Gestational hypertension without proteinuria, *n* (%)	8 (5)	39 (7)	0.10
Pre-eclampsia, *n* (%)	7 (4)	18 (3)	0.46
Type of delivery, *n* (%)			0.034
Vaginal	123 (76)	480 (84)	
Elective caesarean section	14 (9)	42 (7)	
Urgent caesarean section	25 (15)	50 (9)	
One minute Apgar score, mean (SD)	8.4 (1.5)	8.6 (1.2)	0.10
NICU treatment*, *n* (%)	34 (21)	66 (11)	0.002
Antibiotics after delivery*, *n* (%)	17 (10)	35 (6)	0.052
Gestational age, weeks, mean (SD)	38.9 (2.5)	39.6 (1.8)	< 0.001
< 28.0, *n* (%)	2 (1)	2 (0)	
28.0–31.9, *n* (%)	3 (2)	1 (0)	
32.0–33.9, *n* (%)	0 (0)	4 (1)	
34.0–36.9, *n* (%)	13 (8)	23 (4)	
≥ 37.0, *n* (%)	145 (89)	550 (95)	
Boy, *n* (%)	87 (53)	292 (50)	0.49
Birth weight, g, mean (SD)			
Girl	3153 (622)	3439 (526)	< 0.001
Boy	3442 (702)	3554 (559)	0.12
Birth weight, *Z* score, mean (SD)	− 0.00 (1.00)	0.00 (1.00)	0.99
Macrosomia, birthweight ≥ 4500 g, *n* (%)	3 (2)	11 (2)	0.99
Birth length, cm, mean (SD)			
Girl	48.5 (3.5)	49.6 (2.6)	0.002
Boy	50.1 (2.8)	50.6 (2.6)	0.18
Ponderal index, kg/m^3^, mean (SD)	27.4 (2.9)	27.6 (2.6)	0.40

*before age of seven days

*SLE *systemic lupus erythematosus, *n *number, *SD *standard deviation, *g *gram, *cm *centimetre, *m*^*3*^ cubic metre, *NICU *care at neonatal intensive care unit

Altogether 40% of SLE mothers had purchased some anti-rheumatic medication during their pregnancy. Hydroxychloroquine (ATC code P01BA02) was purchased by 30% of the SLE mothers. Purchases of HCQ in SLE patients were not significantly associated with gestational age, birth weight or incidence of pre-eclampsia. Glucocorticoids (ATC code H02) were purchased by 27% of SLE patients and azathioprine (ATC code L04AX01) and cyclosporine (ATC code L04AD) by 7% and 1% of the SLE patients, respectively. Antihypertensive agents were purchased by 3% of SLE mothers during pregnancy compared to 1% of control mothers (*p* = 0.058).

Table 3 displays the occurrence of congenital malformations in the deliveries of SLE mothers (*n* = 8) and in their controls (*n* = 25). No differences in the incidence of congenital malformations were observed between the infants of mothers with SLE and control mothers. Purchased anti-rheumatic medication during pregnancy in those SLE mothers whose infants had congenital malformation were as follows: azathioprine in one, glucocorticoid in 4, hydroxychloroquine in 4 SLE mothers and four of them had no anti-rheumatic medication.

**Table 3:** Congenital malformations in the deliveries of SLE patients and their controls in 2000–2018, in Finland

Congenital malformation with ICD-10-code class	SLE *n* = 163 *n* (%)	Control *n* = 580 *n* (%)	*p* value
At least one	8 (5)	25 (4)	0.74
More than one	1 (1)	10 (2)	0.47
Eye, ear, face and neck, Q10–18	0 (0)	2 (0)	0.99
Cardiovascular, Q20–28	4 (2)	13 (2)	0.77
Cleft palate or lip, Q35–37	1 (1)	0 (0)	0.22
Digestive system, Q38–45	0 (0)	3 (1)	0.98
Genital, Q50–56	0 (0)	1 (0)	0.97
Urogenital, Q60–64	3 (2)	2 (0)	0.073
Musculoskeletal, Q65–79	0 (0)	6 (1)	0.35
Others, Q80–89	0 (0)	1 (0)	0.97
Chromosomal, Q90–99	0 (0)	2 (0)	0.99

*SLE *systemic lupus erythematosus, *ICD– 10 code *International classification of disease- 10th revision code, *n *number, *%* percentage

## Discussion

The study confirms the view that in SLE patients the course of pregnancy differs from the control population. Moreover, in this study, the gestational ages of infants born to SLE mothers were younger compared with those born to control mothers. There were more urgent Caesarian sections, and the need for NICU treatment was greater, in infants born to SLE mothers than in the controls. However, the overall picture of SLE mothers’ pregnancies was positive, and no new safety concerns were identified. Contrary to expectations, the incidence of pre-eclampsia appeared to be similar in both groups.

There is a large volume of published studies describing that preterm birth and small gestational age are common complications in SLE pregnancies [16, 20–25]. In many cases, these are attributed to growth problems during pregnancy. The background can be associated with many things, such as maternal smoking (regardless of SLE), high blood pressure, chronic kidney disease, antiphospholipid antibodies, disease-related characteristics and use of drugs [4, 26, 27]. Commonly, all these predisposing things can cause placental dysfunction by several different mechanisms such as inadequate perfusion, thrombosis or uncontrolled activation of complement [18]. In the present study, the distribution of gestational age was similar to the existent literature [16, 20–23]. Few infants were born prematurely (gestation less than 37 weeks) in both groups. Altogether, 11% of the infants of SLE mothers were born prematurely compared to 5% of infants born to controls. The proportions of prematurity were smaller than in the previous studies from China, Taiwan, Denmark, Canada and Sweden: 14–35% and 7–13%, in SLE and control pregnancies, respectively [16, 20–23].

In Finland, Caesarian sections are performed only when the mother´s or foetus´s condition is unstable, thus it is likely that the abovementioned challenges in SLE pregnancies further led to the increase in urgent Caesarian sections [11, 21, 24]. In the present study, approximately a quarter of the deliveries were Caesarian sections and most of them were urgent in nature. Wu et al. have reported Caesarian sections in 85% of SLE deliveries compared to 56% of non-SLE control deliveries [16]. Of all SLE deliveries in that study, 8% were emergency Caesarian sections. The vast disparity between the percentages of Caesarian sections between this and present studies most probably reflects differences in health care systems and the treatment methods used and indicates that the results between different studies may not be comparable as such [16]. In turn, urgent Caesarian sections and the circumstances leading or related to them, especially respiratory distress syndrome, result in numerous NICU treatment episodes [28]. In the present study, one-fifth of the neonates needed NICU treatment. Elsewhere, the percentages of need for NICU treatment after SLE deliveries have varied between 16 and 52% and the risk for NICU treatment has been estimated to be 2–3.5 times higher in neonates born to SLE mothers compared with the background population [6, 16, 22, 24, 29].

Pre-eclampsia is a possibly life-threatening condition that occurs in 3–5% of all pregnancies [30]. In Finland, the prevalence of pre-eclampsia has been estimated to be between 2 and 3% of pregnancies [31, 32]. The primary treatment option is delivery [30]. Data from several sources have identified an increased incidence of pre-eclampsia associated with SLE pregnancies [6, 15, 22, 23, 25]. The likelihood of pre-eclampsia in SLE mothers has been estimated to be 2–4 times higher compared to the background population [6, 15, 22, 25, 33, 34]. Nevertheless, in the present study, there was no difference in the occurrence of pre-eclampsia between SLE mothers and their controls. Since this result from the Medical Birth Register was unexpected, it was further confirmed by the Care Register, and the result remained the same. There is evidence showing that maternal hypertension raises the likelihood of complications in lupus pregnancies [14, 35]. Thus, theoretically use of antihypertensive agents could have a protective influence on further complications. In the present study, SLE mothers more frequently purchased antihypertensive medication than their control counterparts, but the difference in purchases of antihypertensive medication was not significant. Still, there is a Japanese study on lupus pregnancies showing no increased level of pre-eclampsia in SLE pregnancies compared to figures earlier reported in the population (6% versus 10%) [36], and a Danish study detecting an increased but not significant trend of pre-eclampsia in SLE versus control pregnancies [21]. Further, the differential diagnosis between lupus nephritis at gestation and pre-eclampsia in SLE mothers is not always straightforward, and in our register material, we depend on the clinicians´ ability to make the correct diagnosis without the ability to check the clinical data afterwards. Thus, it is possible that, for example, the prevalence of pre-eclampsia in SLE is an underestimation.

Several studies have found HCQ to be beneficial during pregnancy [37–43]. It seems that HCQ is related to lower disease activity during SLE pregnancy [37]. Saavedra et al. [39] showed that the likelihood of pre-eclampsia was lower in SLE patients using antimalarial drugs during pregnancy. HCQ has the ability to cross the placenta [44]. It has been shown that HCQ inhibits lysosomal and extralysosomal toll-like receptors (TLRs), which block systemic inflammation in pre-eclampsia and in recurrent miscarriage [42]. Moreover, Abd Rahman et al. [38] showed that the use of HCQ was related to a longer duration of pregnancy, higher birth weight, lower incidence of gestational diabetes and had a protective effect against high blood pressure. Also, the prevalence of congenital conduction defects has been shown to be lower in HCQ users [40]. The safety profile of antimalarial drugs during pregnancy has been shown to be good, and they are not associated with prematurity, low birth weight or increased occurrence of congenital malformations [43]. Despite the fact that HCQ is recommended for all pregnant SLE patients [13], the use of HCQ was at a lower level than expected in the present cohort. This might reflect low adherence to medication during the pregnancy. The method used shows the prescribed and purchased drugs, which is a closer estimation of the reality of drug use than just evaluating the drugs prescribed. Although there are robust findings demonstrating the benefits of HCQ [37–40], not all pregnant SLE patients seem to comply with taking the medicine. Due to the limited use of HCQ in our cohort, it is impossible to draw conclusions on the relevance of the medication to pregnancy in SLE. Similar proportions of HCQ use (37.7%) in 2015 were reported in a US study, which was based on a Medicaid database [41]. On the other hand, in contrast to the present results, a Portuguese retrospective study from 1993 to 2019 reported a high percentage of HCQ use (64%) in pregnant SLE mothers, to whom counselling before gestation was given in 87% of pregnancies [45].

As a whole, the pregnant SLE mothers in the present study had limited anti-rheumatic drug use: a quarter of the SLE patients in the present study used glucocorticoids, and azathioprine was used by only 7% of pregnant SLE mothers. A Canadian population-based study demonstrated a large proportion of discontinuations of SLE drugs before and during pregnancies. The proportions of drugs used for SLE were similar to the present study. Glucocorticoids, antimalarials and azathioprine were used by 20%, 24% and 8% of the pregnant SLE patients, respectively [46]. At the same time, there is a growing body of literature showing that active disease is one of the major risk factors antedating complications during pregnancy in SLE patients [35, 47]. The present result draws our attention to the importance of considering proper counselling of patients during pregnancy.

There is still uncertainty regarding whether a mother’s SLE predisposes the foetus to congenital malformations. A meta-analysis of 11 studies published in 2001–2016 reported over two times increased odds for congenital defects in SLE pregnancies [29]. A recent nationwide Korean study showed a significantly increased risk for congenital deficiencies in infants born to SLE mothers compared to those born to population controls. The differences in malformations were especially seen in the region of the eye, ear, face and neck, and moreover, in neurological, circulatory and musculoskeletal systems [48]. In the present study, there was no significant difference in the occurrence of major congenital malformations between SLE pregnancies and those of controls. Likewise, a Canadian study found no increase in congenital defects among SLE pregnancies in 1998–2009 compared to matched population controls [22].

In the present study, a higher percentage of SLE mothers were primiparous than control mothers. It is likely that if the previous pregnancy had been complicated, SLE mothers either postpone or abandon the idea of a subsequent pregnancy. This hypothesis is supported by a recent large register-based study showing that the mean number of pregnancies per mother was significantly lower in SLE mothers than in controls [25]. Consistently, a Korean study showed that the pregnancy rate was about a third lower in SLE women than in the background population [15].

As the registered data of the present study were nationwide, there was no selection bias. On the other hand, milder cases of SLE were also included in the pregnancy data. In this study, it was possible to combine reliable data from different registers, expanding the overall picture of the course of pregnancy in SLE patients. The selected study method of a case–control setting increases the value of the study. A major limitation of the study is the lack of clinical data before and during pregnancies. It is possible that not all information is supplied in the registries. As the data are based on the babies who were born alive, it was not possible to analyse data on miscarriages or stillbirths.

Despite the fact that SLE mothers face several possible challenges during their pregnancy, the present results were rather encouraging. No new safety issues were identified in the SLE pregnancies, and the overall picture of them did not differ that much from the pregnancies of the controls. The current recommendations for SLE pregnancies, however, were not strictly followed and better results are possible to achieve [13, 49]. The theoretical basis of the impacts of pregnancy on SLE and vice versa is poorly understood. Much of the recent research has focused on evaluating the activity of disease during pregnancy [1, 50]. Further work is required to establish the nature of immunological processes in pregnancies of SLE mothers. Extending clinical SLE registries to clinicians’ daily work and combining bio-bank data with clinical data would improve knowledge in this field.
